# Practicing Social Isolation During a Pandemic in Brazil: A Description of Psychosocial Characteristics and Traits of Personality During COVID-19 Lockout

**DOI:** 10.3389/fsoc.2021.615232

**Published:** 2021-05-10

**Authors:** Daniela Sacramento Zanini, Evandro Morais Peixoto, Josemberg Moura de Andrade, Lucia Tramonte

**Affiliations:** ^1^ Department of Psychology, Pontifical Catholic University of Goiás, Goiania, Brazil; ^2^ Department of Psychology, University of São Francisco USF, Bragança Paulista, Brazil; ^3^ Department of Social and Work Psychology, University of Brasília, Brasília, Brazil; ^4^ Department of Sociology, University of New Brunswick, New Brunswick, NB, Canada

**Keywords:** pandemic, social isolation, psychological characteristics, personality traits, pro-social behavior, social support

## Abstract

The experience of the pandemic caused by the Coronavirus and the consequent disease triggered by it (COVID-19) brought to light fragilities that have been long overlooked by the scientific community and by various political and social institutions. The pandemic also brought to the fore certain social practices resulting from individual behaviors, such as wearing a mask and practicing social isolation. It demonstrated the need for social commitment and pro-social behaviors if societies are to respond successfully. The purpose of this article is to evaluate psychological and sociodemographic characteristics associated with compliance or noncompliance of individuals with these practices in two different phases of the pandemic experience in Brazil: in the first month and after three months. Participants for the first phase of the study were recruited through advertisements in the media and social networks. 1,914 individuals aged between 14 and 81 years agreed to participate, 78.2% of these were women, from 25 Federative Units in Brazil. In the second phase, 761 individuals who participated in the first phase, were reassessed. The authors used the following instruments for data collection: a standardized questionnaire collecting information of sociodemographic characteristics and dynamics of social isolation; the Kessler Psychological Distress Scale; the Life Satisfaction Scale; the Positive and Negative Affections Scale; and the Reduced Personality Markers and Stress Mindset Scale. All instruments used presented evidence of validity and adequate reliability indexes. The comparison of categorical exploratory variables with motives for following social isolation protocols was performed using Pearson’s Chi-square, and the comparison of continuous exploratory variables was performed using the Mann-Whitney test. Covariance Analysis was performed using as covariates those that showed significance/effect on isolation in previous analyses. The results showed that respondents practicing social isolation to comply with governmental recommendations had lower scores on the scales of neuroticism and conscientiousness. They reported also less stress, anxiety, and depression, and less general distress. Overall, these respondents also displayed more positive affect, and tended to reframe stress in a more positive way than others. These preliminary results describe the psychological characteristics of individuals and their associations with social behaviors in a period of collective stress and high social risk.

## Introduction

The disease caused by Coronavirus Disease 2019 (COVID-19) was first identified in December 2019 in the city of Wuhan, China, and in January 2020 the World Health Organization (WHO) declared it a public health emergency of international interest (Maia and Dias, 2020). The pandemic can be identified as a stress-inducing social reality and it requires appropriate individual behavior to contain its worst effects. In this way it can be describe as a stressful live event that demanded coping strategies to face it (Lazarus and Folkman, 1984). As such, it has brought to light individual and social fragilities that have been long overlooked by the scientific community and social institutions. Among the most prominent of these are individual attitudes and behaviors that can be described as anti-social; the difficulty of acting locally on what are global problems; and the permeability of boarders and the interconnectedness of individuals and groups when social isolation is required. The pandemic has showcased the social consequences of individual behaviors, such as wearing a mask or adopting practices of social isolation, as well as the need for the social commitment and pro-social behaviors that controlling this pandemic requires.

In terms of public health, slowing the spread of the virus has challenged current social practices and behaviors which have tended to reduce compliance to the new social rules now required to slow the spread of the Coronavirus (Fischer et al., 2020). This manuscript presents a study of the association of certain personality and psychosocial aspects with social behavior and commitment to social isolation to control the pandemic in Brazil.

### The Pandemic as a Social Science Challenge

Since early 2020, there has been an abundance of research on Covid-19 pandemic mainly from medical and epidemiological disciplines (Ward, 2020). However, there is a critical need to have an interdisciplinary conversation, both theoretically and empirically, on the psychosocial and sociological consequences of the pandemic experience (Ferreira et al., 2020).

The socio-epidemiological literature on pandemics documented the many challenges that governments and public health institutions faced in encouraging the population to practice and sustain restrictive behaviors. For example, (van der Weerd et al., 2011) described the social resources used by public health during the early stages of the influenza virus (H1N1) pandemic, when there was still no vaccine or drug treatment available. At that time, preventative practices such as wearing a mask, maintain proper hygiene, and social distancing to reduce the contagion were seldom adopted by the public. Importantly, risk perception and the degree of confidence in the information transmitted by health authorities significantly influenced the personal decision to adopt protective measures.

Giubilini et al. (2018) pointed to the benefits of restrictive behaviors such as quarantine and social isolation in tackling Ebola infections between 2014 and 2015 in West Africa, as well as the social challenges for their implementation. The authors justified the application of coercive and compulsory measures by the State as a way of controlling infections with the potential to harm the whole of society, but they also reminded us of the need for a compelling justification for the implementation of such actions. These discussions become even more relevant as individual protective measures cannot easily be implemented entirely by coercion in democratic societies (Clark A. et al., 2020).

In the context of Covid-19 pandemic, all countries committed to the goal of reducing the spread of the coronavirus, but some have been more successful than others. Researchers have identified differences in personality traits and social behaviors that positively or negatively influence whether an individual will engage in the social health practices needed to contain Covid-19 (Zajenkowski et al., 2020). For example, people who have lower levels of “agreeableness” (sense of cooperation and social Harmony) and “conscientiousness” (self control and practicality), or who manifest psychopathological personality traits such as machiavellism, narcissism or psychopathy (Blagov, 2020), or antisocial traits represented by lack of empathy, callousness, deceitfulness and risk-taking (Miguel et al., 2020) tend to show lower levels of commitment to practices controlling the contagion.

Although theoretically, all five major personality traits can relate differently to the restrictive behavior demanded to face Covid-19, the results from a Polish sample showed that only agreeableness predicted compliance to the Covid-19 social and individual restrictions (Zajenkowski et al., 2020). Also, individuals with different personality traits perceived risk differently during the Covid-19 pandemic.

A large body of research is growing to address the psychosocial consequences of the pandemic and the stressors associated with measures of contagion control, with special emphasis on social isolation. These include increased levels of anxiety, depression and stress, confusion, anger, and even post-traumatic stress (Brooks et al., 2020; Maia and Dias, 2020). The consequences of social isolation can also be different depending on personality traits and individuals' characteristics (Zajenkowski, et al., 2020). The perception of stress is worsened by feelings of ignorance about the disease, paired with confusing, inconsistent messages from the media, as well as the perception of risk for oneself or one’s group of origin (Brooks et al., 2020; Clark C. et al., 2020; Zajenkowski, et al., 2020).

This study has been designed to aid researchers, health managers, and government officials in understanding the psychological and social factors that can contribute to the population’s engagement in behaviors needed to reduce the spread of the disease. This knowledge can help authorities to better implement public health measures as well as to encourage different groups of the population to adhere to these public health policies.

### The Context of Brazil

These challenges are greater in a country like Brazil that is multiethnic, geographically diverse, and with a significant degree of social inequality (Instituto Brasileiro de Geografia e Estatística (IBGE), 2018). In Brazil, social isolation was presented as a containment measure to prevent the spread of Covid-19 through the national Quarantine Law (Law No. 13,979; Diário Oficial da União–DOU, 2020). However, local governments were given autonomy in making decisions about the best strategies to face the pandemic according to the epidemiological profile of each city or Federative Unit (Moreira, 2020). Lack of explicit guidance on the part of the federal government, exacerbated by turnover of health ministers, left local political leaders uncertain about what measures to adopted and enforce (Aquino et al., 2020). Inconsistent measures and guidelines from government officials at different jurisdictional levels, and from health authorities fueled people’s sense of confusion, uncertainty, ignorance and mistrust, raising even further the stress resulting from the experience of the Covid-19 pandemic. People were left to decide on their own if and how to protect themselves and others from the spread of the Covid-19, there seemed to be no consistent policy to confront the pandemic (Teixeira et al., 2020). That created a sense of solidarism between different groups and organizations to face the pandemic in Brazil.

This manuscript reports the preliminary results of a study designed to investigate psychological and sociodemographic traits associated with attitudes and behaviors relating to social isolation, social distancing, and mark-wearing in two different phases of the pandemic experience in Brazil: in the first month (April 2020) and after three months (May to June 2020). This paper also describes the psychological and sociodemographic profiles of people who reported practicing social isolation for different reasons: 1) as a social commitment and pro-social behaviors, such as to avoid infecting others, 2) to follow the authorities’ recommendation, or 3) for individualistic reasons, particularly to avoid getting infected. The samples in the two phases of this study have comparable sociodemographic characteristics and can be used to compare over the course of the pandemic the association between psychological profiles and the social practices of mask-wearing and practice isolation.

## Methods

### Study Design

This study is correlational and is designed to collect data from Brazilian adults age 14–81 years old, in two different months of the pandemic.

Phase 1 includes participants who filled out an online questionnaire during the month of April, 2020, approximately one month after the start of the pandemic in Brazil. Phase 2 started on May and continued until June, 2020, three months into the pandemic. Some of the participants in phase 2 also participated in phase 1. For this preliminary analysis, we treated the two phases as if independent. So, this manuscript reports preliminary results from the first two available phases, April 2020 and June 2020.

### Participants

In the first month of the pandemic in Brazil (Phase 1), 1,914 individuals answered an online questionnaire. We focused on 1,842 of them, 78.2% women and 21.8% men, those who were all practicing social isolation. Their age ranged between 14 and 81 years (M = 34.91, SD = 13.70). Three percent of them were from the North; 29.7% from the Northeast; 28.3% from the Midwest; 30.7% from the Southeast and 8.3% from the South. About half of the respondents were single (51.2%), about 30% married (30.3%), 8% in a stable relationship (8.3%), 8% divorced (8%), and 1% were widowed (1.1%) or other (1, 2%), respectively.

In the second phase of the study corresponding to the third month of the pandemic in Brazil, 761 individuals answered the questionnaire; only 452 (82.5% were women and 17.5% were men) were practicing social isolation. The age of the sample in the second phase ranged between 17 and 77 years (M = 34.01, SD = 12.97); 7.1% were from the North, 31.2% from the Northeast, 29.2% from the Midwest, 29.6% from the Southeast and 2.9% from the South. Most of the respondents were single (55.8%), followed by married (26.5%), in a stable relationship (10.4%), divorced (5.8%), widowed (0.9%) and others (0.7%).

For both phases, the following inclusion criteria were considered: agreeing to be part of the study by accepting the Free and Informed Consent Term, as well as answering all questions in the questionnaire.

### Instruments

The online questionnaire contained two sections. Section one collected information on sociodemographic characteristics and practices of social isolation; section two administered several psychological scales. All psychological scales or measures presented evidence of validity based on the internal structure and adequate reliability indexes.

#### Section One: Sociodemographic Characteristics and Practices of Social Isolation

This section was developed to collected sociodemographic information such as age, sex, marital status, occupational sector and family income level of the respondents. Other questions collected are presence of people at high risk or children in the family unit or household; levels of activity during social isolation; and information on compliance to social isolation, including why respondents practiced social isolation. In relation to this last question, participants were asked for the principal motive for socially isolating. They were given three options: 1) comply with governmental recommendations; 2) avoid infecting others; and 3) avoid getting infected themselves.

#### Section Two–Psychological Scales

Section Two of the Questionnaire Contained Six Psychological Scales, Described Here Below:

##### Reduced personality markers

Originally, markers for personality assessment in the *Big Five Model* were developed by Hutz et al. (1998). It is an instrument composed of 64 adjectives divided into five subscales: neuroticism (low emotional stability, referring to a greater tendency to experience negative emotions), extraversion (sociability or engagement in the outside world), openness to experiences (interest in different areas, tending to originality and imagination), agreeableness (sense of cooperation and social harmony) and conscientiousness (self control and practicality). Participants should complement the statement “I am a person ...”. The response scale is a five-point *Likert*, with the following extremes: 1 = “Strongly disagree” and 5 = “Strongly agree”. Subsequently, Hauck Filho et al. (2012) developed a reduced version composed of 25 adjectives whose response procedures follow those of the original scale.

The authors reported satisfactory evidence of validity and accuracy indexes using Cronbach's alpha between 0.61 (opening to experience) and 0.83 (extroversion).

### Stress Mindset Scale

Developed by Crum et al. (2013), SMS assesses the beliefs that a person has about the consequences that stress can have on their performance and growth. The scale consists of eight items, four positive (e.g., “The effects of stress are positive and can be useful”) and four negative (e.g., “Experiencing stress exhausts my health and vitality”), answered using the *Likert* type, where responses range from 0 = “Strongly disagree” to 4 = “Strongly agree”. An adaptation study and evaluation of the evidence of SMS validity for the Brazilian context showed the adequacy of a unidimentionality, as well as good reliability indicators, Crobach’s alpha equal to 0.868 and McDonald's Omega equal to 0.869 (Peixoto et al., 2019). To compose the final score in stress mindset, the negative items must be inverted, and all the items added up; higher values of these scores indicate higher levels of positive mindset.

### Depression, Anxiety and Stress Scale

The Depression, Anxiety and Stress scale (DASS-21) was developed by (Lovibond and Lovibond, 1995), assesses these three constructs using three factors of the same name, composed of seven items each, totaling 21 items on the scale. The response scale is a four-point Likert scale, ranging from 0 (“did not apply at all”) to 3 (“applied a lot or most of the time”). In Brazil, DASS-21 was adapted, and its psychometric properties were evaluated in several regions of the country with samples of adolescents (Patias et al., 2016; Silva et al., 2016) with results that assure the three-factor structure and good reliability indicators between 0.83 and 0.96.

### Kessler Psychological Distress Scale (K10)

The translation and adaptation of the k10 scale was carried out to assess psychological distress in the Brazilian population. The instrument presented evidence of validity based on the internal structure from a single-factor structure with a Cronbach's alpha reliability index of 0.90. This instrument has 10 self-report items that assess the level of emotional distress related to experiencing stress in the last 30 days. Items are answered using a five-point *Likert* scale with the following extreme points 1 = “no time” and 5 = “all the time”. Higher scores mean a higher level of psychological distress and psychological distress (Andrews and Slade, 2001).

### Life Satisfaction Scale

Developed by Diener et al. (1985), the LSS assesses cognitive aspects of subjective well-being and is considered the gold standard for assessing this construct (Diener, 2000). This scale has five self-report items that assess the level of satisfaction with the respondent's life using a seven-point *Likert*-type response scale. The Brazilian version was adapted and validated by (Zanon et al., 2013a) and their adaptation studies demonstrate good evidence of accuracy and validity and preliminary standards (Hutz et al., 2014).

### Positive and Negative Affections Scale

Originally developed by (Watson et al., 1988), PANAS assesses the emotional components of subjective well-being through positive (PA) and negative affections (NA). The scale consists of 20 words that express emotions, ten relating to positive affections and 10 relating to negative affections that are answered using a five-point *Likert* scale (1 = “not at all” and 5 = “extremely”). In Brazil, the scale was adapted by Zanon, Bastianello, Pacico, and Hutz, in 2013, presenting studies that demonstrate good precision, evidence of factorial validity and preliminary norms (Zanon et al., 2013b).

### Procedure

Participants were recruited through advertisements in the media and social networks (*Whatsapp*, *Facebook*, *Instagram* etc.). The invitation to voluntary participation was attested by accepting the Terms of Free and Informed Consent, which guaranteed anonymity and the possibility of withdrawing at any stage of the survey. The Terms of Free and Informed Consent provided the contacts of the research team for further information on the study or if the respondents experienced any unwanted complications resulting from participation in the study.

Participants responded to the questionnaire online, using the GoogleForms software.

### Data Analysis

We conducted descriptive and inferential statistical analyses using the statistical software package SPSS 26.0. As a first step, we described the sociodemographic profile of individuals with different motivations and rationales for their adherence to practices of social isolation. We then analyzed the relation between the main reason for socially isolating and other variables.

For categorical variables, we presented means of absolute frequency (n) and relative frequency (%); for continuous variables, we reported means and standard deviations. Specifically, for the parametric test - Ancova - the normality of the variables was tested using the Kolmogorov-Smirnov test. The results indicated normality of the continuous variables. We measured the association between categorical exploratory variables and the principal motive for socially isolating using Pearson’s Chi-square and Post-hoc Chi-square tests with Bonferroni correction when significant differences were found in contingencies greater than 2 × 2, as suggested by (MacDonald and Robert, 2000).

The comparison of continuous exploratory variables with the principal motive for socially isolating was performed using the Mann-Whitney test. We isolated the effect of social isolation on well-being (positive and negative affect), life satisfaction, psychological distress (K10) and personality traits, by applying Covariance Analysis and selecting as covariates those that showed significant effect on isolation in previous analysis (sex, marital status, profession, schooling, family income). For all the analyses, we adopted the 95% confidence interval (*p* < 0.05).

## Results

Tables 1, 2 describe the sociodemographic characteristics of the sample by social isolation according to principal motive for socially isolating in phase 1 and 2, respectively. Three types of reasons for adhering to social isolation were considered, namely: 1) Comply with governmental recommendations, 2) Avoid infecting others, and 3) Avoid getting infected. The results of phase 1 are shown in Table 1.

**TABLE 1:** Sociodemographic characteristics of the sample according to reasons for adherence to social isolation on phase 1 by means (age) and frequency (sex, marital status, profession, educational level, family income, risk group) (*n* = 1,437).

	Motives to practice social isolation			Total	*p*
	Comply with governmental recommendations	Avoid infecting others	Avoid getting infected		
Age (Mean ± Standard deviation)	39.17 ± 15.18	30.94 ± 11,19	36.34 ± 13.89	34.89 ± 13.69	< 0.001^**^
Sex					
Female	402 (75.1)	606 (78.1)	429 (81.4)	1437 (78.2)	0.05^*^
Male	133 (24.9)	170 (21.9)	98 (18.6)	401 (21.8)	
Marital status					
Married	181 (33.8)^a^	189 (24.4)^b^	185 (35.1)^a^	555 (30.2)	0.001^*^
Divorced	52 (9.7)	50 (6.4)	45 (8.5)	147 (8.0)	
Single	236 (44.1)^b^	477 (61.5)^a^	229 (43.5)^b^	942 (51.3)	
Stable relationship	51 (9.5)	47 (6.1)	54 (10.2)	152 (8.3)	
Widower	11 (2.1)	3 (0.4)	6 (1.1)	20 (1.1)	
Others	4 (0.7)	10 (1.3)	8 (1.5)	22 (1.2)	
Profession					
Retired	43 (8.0)^a^	10 (1.3)^b^	28 (5.3)^a^	81 (4.4)	0.001^*^
Private sector	86 (16.1)^b^	180 (23.2)^a^	97 (18.4)^b^	363 (19.7)	
Public sector	132 (24.7)	173 (22.3)	143 (27.1)	448 (24.4)	
Self employed	99 (18.5)	118 (15.2)	87 (16.5)	304 (16.5)	
Unemployed	135 (25.2)	246 (31.7)	145 (27.5)	526 (28.6)	
Educational level	40 (7.5)	49 (6.3)	27 (5.1)	116 (6.3)	
High school					
UnderGraduate level	40 (7.5)	43 (5.5)	21 (4.0)	104 (5.7)	**<** 0.001^*^
Graduate level	197 (36.8)^b^	387 (49.9)^a^	227 (43.1)^b^	811 (44.1)	
Family income	3 (0.6)	2 (0.3)	3 (0.6)	8 (0.4)	
Less than minimum wage	295 (55.1)^a^	344 (44.3)^b^	276 (52.4)^a^	915 (49.8)	
Between 1 and 3 minimum wage					
Between 3 and 5 minimum wage	36 (6.7)	38 (4.9)	24 (4.6)	98 (5.3)	0.29*
Between 5 and 10 minimum wage	116 (21.7)	152 (19.6)	117 (22.2)	385 (20.9)	
Between 10 and 20 minimum wage	88 (16.4)	127 (16.4)	106 (20.1)	321 (17.5)	
More than 20 minimum wage	108 (20.2)	195 (25.1)	110 (20.9)	413 (22.5)	
Risk group					
No	392 (73.3)	653 (84.1)	355 (67.4)	1400 (76.2)	
Yes	143 (26.7)^a^	123 (15.9)^b^	172 (32.6)^a^	438 (23.8)	**<**0.001*

^*^Pearson Qui-squared (n, absolute frequency; %, relative frequency). **Kruskal-Wallis test (mean ± standard deviation).

^a^ Equal letters on the same line indicate no significant difference in post hoc.

^b^ Equal letters on the same line indicate no significant difference in post hoc.

**TABLE 2:** Sociodemographic characteristics of the sample according to reasons for adherence to social isolation on phase 2 by means (age) and frequency (sex, marital status, profession, educational level, family income, risk group) (*n* = 452).

	Motives for practicing social isolation			Total	*p*
	Comply with governmental recommendations	Avoid infecting others	Avoid getting infected				
Age (Mean ± standard deviation)	38.73 ± 14.73	30.48 ± 11.35	34.95 ± 12.53	34.01 ± 12.97	0.001**
Sex					
Female	81 (82.7)	151 (84.8)	141 (80.1)	373 (82.5)	0.50^*^
Male	17 (17.3)	27 (15.2)	35 (19.9)	79 (17.5)	
Marital status					
Married	32 (32.7)	36 (20.2)	52 (29.5)	120 (26.5)	0.04^*^
Divorced	10 (10.2)^a^	5 (2.8)^b^	11 (6.3)^b^	26 (5.8)	
Single	44 (44.9)^b^	118 (66.3)^a^	90 (51.1)^b^	252 (55.8)		
Stable relationship	11 (11.2)	16 (9.0)	20 (11.4)	47 (10.4)		
Widower	1 (1.0)	2 (1.1)	1 (0.6)	4 (0.9)		
Others	0 (0.0)	1 (0.6)	2 (1.1)	3 (0.7)		
Profession					
Retired	5 (5.1)	1 (0.6)	8 (4.5)	14 (3.1)	0.002^*^
Private sector	13 (13.3)	35 (19.7)	22 (12.5)	70 (15.5)	
Public sector	40 (40.8)^a^	42 (23.6)^b^	68 (38.6)^a^	150 (33.2)		
Self employed	19 (19.4)	36 (20.2)	26 (14.8)	81 (17.9)		
Unemployed	21 (21.4)^b^	64 (36.0)^a^	52 (29.5)^b^	137 (30.3)		
Educational level					
High school	0 (0.0)	3 (1.7)	5 (2.8)	8 (1.8)	0.01^*^
UnderGraduate level	30 (30.6)^b^	86 (48.3)^a^	68 (38.6)^b^	184 (40.7)	
Graduate level	68 (69.4)^a^	89 (50.0)^b^	103 (58.5)^b^	260 (57.5)		
Family income					
Less than minimum wage	5 (5.1)	9 (5.1)	12 (6.8)	26 (5.8)	0.93^*^
Between 1 and 3 minimum wage	19 (19.4)	29 (16.3)	30 (17.0)	78 (17.3)	
Between 3 and 5 minimum wage	20 (20.4)	38 (21.3)	43 (24.4)	101 (22.3)		
Between 5 and 10 minimum wage	28 (28.6)	53 (29.8)	51 (29.0)	132 (29.2)		
Between 10 and 20 minimum wage	18 (18.4)	33 (18.5)	32 (18.2)	83 (18.4)		
More than 20 minimum wage	8 (8.2)	16 (9.0)	8 (4.5)	32 (7.1)		
Risk group					
No	68 (69.4)	154 (86.5)	118 (67.0)	340 (75.2)	
Yes	30 (30.6)^a^	24 (13.5)^b^	58 (33.0)^a^	112 (24.8)	0.001^*^

^*^Pearson Qui-squared (n, absolute frequency; %, relative frequency). ^**^Kruskal-Wallis test (mean ± standard deviation).

^a^ Equal letters on the same line indicate no significant difference in post hoc.

^b^ Equal letters on the same line indicate no significant difference in post hoc.

Except for sex and family income, in phase 1 there were significant differences in participants socioeconomic profiles that were associated with their motives for practicing social isolation. Those who practiced social isolation to follow the authorities’ recommendation were older (M = 39.17, SD = 15.18) than those who practiced social isolation for other reasons. Those who did not want to infect others were more often unmarried than married, active in the labor force of the private sector, and educated at the “undergraduate level”. People who declared that they were at risk reported more often to practicing social isolation to comply with governmental recommendation and to protect themselves.

Table 2 summarizes the same information but for phase 2, showing significant differences between the principal motive for socially isolating and some sociodemographic characteristics, except again for sex and family income as well as, now, for geographic region.

In phase 2 people who practiced social isolation to comply with governmental recommendation were older and more often divorced. The unmarried were more prone to report practicing social isolation to avoid infecting others.

Different from phase 1, on phase 2 the public workers were less motivated to social isolate to avoid infecting others. However, as in phase 1, in phase 2 unemployed persons, more than other groups, practiced social isolation to avoid infecting others. This group had more individuals with a “undergraduate level” of education. People with a “graduate level of education”, more likely practiced social isolation. Also, people in the “at risk” group reported more often to practicing social isolation to comply with governmental recommendation and to protect themselves. Employees in the public sector reported more frequently practicing social isolation to comply with governmental recommendation.

Phase 1 had more than three times more participants than phase 2. The sex and age distributions were similar in both phases.

Table 3, 4 show the psychological characteristics of the respondents by motives to practice social isolation in phases 1 and 2, respectively.

**TABLE 3:** Personality trait and psychological characteristics according to reasons for adherence to social isolation on phase 1.

	Motives for practicing social isolation			*p* ^*^	*p* ^**^
	Comply with governmental recommendations	Avoid infecting others	Avoid getting infected		
Agreeableness	20.27 ± 2.88	20.16 ± 2.86	19.96 ± 3.13	0.42	0.25
Neuroticism	13.69 ± 4.59^a^	15.50 ± 4.31^b^	15.19 ± 4.35^b^	< 0.001	< 0.001
Extroversion	17.33 ± 3.64	16.65 ± 3.98	17.14 ± 4.19	0.003	0.80
Openness	15.62 ± 3.74	15.50 ± 3.76	15.21 ± 3.73	0.12	0.31
Conscientiousness	21.03 ± 3.07^a^	20.36 ± 3.04^b^	20.70 ± 3.17	< 0.001	0.02
Mindset for stress	10.01 ± 6.24	9.43 ± 6.32	8.85 ± 5.98	0.007	0.008
Stress	7.11 ± 5.02^a^	8.68 ± 4.78^b^	8.16 ± 4.80^b^	< 0.001	< 0.001
Anxiety	4.50 ± 5.10^a^	5.96 ± 5.38^b^	5.60 ± 5.42^b^	< 0.001	< 0.001
Depression	5.76 ± 5.33^a^	7.15 ± 5.65^b^	6.71 ± 5.40^b^	< 0.001	0.001
K-10	23.50 ± 8.67^a^	26.83 ± 8.52^b^	25.60 ± 8.39^c^	< 0.001	0.001
Meaning of life	22.14 ± 6.90	21.91 ± 6.71	21.83 ± 6.62	0.71	0.33
Positive affects	31.21 ± 8.13^a^	28.44 ± 7.59^b^	28.33 ± 7.54^b^	< 0.001	0.001
Negative affects	26.23 ± 9.29^a^	29.18 ± 8.75^b^	28.57 ± 8.80^b^	< 0.001	0.001

^*^Kruskal-Wallis Test. ^**^ANCOVA test.

^a^ Equal letters on the same line indicate no significant difference in post hoc.

^b^ Equal letters on the same line indicate no significant difference in post hoc.

^c^ Equal letters on the same line indicate no significant difference in post hoc.

**TABLE 4:** Psychological characteristics according to reasons for adherence to social isolation on phase 2.

	Motives for practicing social isolation			Total	*p* ^*^	*p* ^**^
	Comply with governmental recommendations	Avoid infecting others	Avoid getting infected			
Mindset for stress	9.74 ± 6.12	10.06 ± 6.03^a^	8.78 ± 5.96^b^	9.49 ± 6.04	0.11	0.02
Stress	8.80 ± 5.38	10.15 ± 5.04	9.68 ± 5.46	9.67 ± 5.29	0.11	0.84
Anxiety	4.41 ± 4.85	5.15 ± 4.68	5.54 ± 5.43	5.14 ± 5.03	0.16	0.25
Depression	6.15 ± 5.10	7.62 ± 5.75	6.60 ± 5.32	6.90 ± 5.47	0.09	0.67
K-10	24.05 ± 8.22	26.49 ± 8.58	25.48 ± 8.73	25.57 ± 8.59	0.09	0.85
Meaning of life	22.96 ± 6.60	22.93 ± 6.41	22.80 ± 6.40	22.88 ± 6.43	0.84	0.42
Positive affects	27.08 ± 7.59	27.25 ± 7.58	27.05 ± 8.00	27.14 ± 7.73	0.91	0.32
Negative affects	26.57 ± 8.37	28.67 ± 8.76	28.14 ± 8.69	28.01 ± 8.67	0.19	0.70

^*^Kruskal-Wallis Test; ^**^ANCOVA.

^a^ Equal letters on the same line indicate no significant difference in post hoc.

^b^ Equal letters on the same line indicate no significant difference in post hoc.

In phase 1, those who practiced social isolation to comply with governmental recommendations presented different mean scores on almost every psychological scale compared to those who practiced social isolation to avoid infecting others or themselves (Table 3), even when controlling for a set of sociodemographic covariates (see significative variables on Table 1). More specifically, people who practiced social isolation to comply with governmental recommendations had lower scores on neuroticism and conscientiousness, had less stress, anxiety, depression, and general distress, more positive affect, less negative affect and tended to see stress in a more positive way than others.

On phase 2, we could not find the difference observed on phase 1 for psychological characteristics. The results are shown below in Table 4.

The only significant difference that we found was on stress mindset when controlling for a set of sociodemographic covariates (see significative variables on Table 1). People who practiced social isolation to avoid infecting others tended to see stress in a more positive way than those who did it to avoid getting infected.

## Discussion

The pandemic presents the deepest public health and economic crisis of our times. Extreme measures have been taken: countries are in lockdown; borders have closed; and individuals were socially isolated for the collective health (Matthewman and Huppatz, 2020). Specifically, in the Brazilian context, the effects of the pandemic were further aggravated by social inequalities and high-income concentration by specific groups in society. Social inequality is a driver of disaggregation and concrete vulnerabilities (Godinho, 2011). Also, Brazil is a large and diverse country with strong spatial heterogeneities in terms of demography, age distribution and access to public health. Considering these inequalities, the Covid-19 pandemic should impact these populations differently (Coelho et al., 2020) and engender different ways of coping with the pandemic and social isolation. Understanding of psychosocial aspects related to social isolation and the behavior of individuals in the context of a distressful event like the pandemic could help produce better public help policies. As a result, this study investigated whether people who reported practicing social isolation for reasons of social commitment such as to avoid infecting others, presented different psychological and sociodemographic profiles than those who were moved by other considerations and rationales, such as to comply with governmental recommendations or to avoid getting infected. More respondents participated in the survey in April than in July, but the two phases (phase 1 and 2) have similar sociodemographic profiles. The results of this study indicate that people who practiced social isolation as a social commitment were younger, mostly single, and with a “undergraduate level” of education. They were more often employed in the private sector (phase 1) and less frequently in the public sector (phase 2) and they perceived themselves as not being in the risk group. This finding opens an interesting scenario of the pandemic in Brazil, where the younger and better educated, with access to scientific information, also displayed greater social commitment and pro social behaviors in face of the pandemic. These same people had higher neuroticism levels and less “conscientiousness” levels compared to those who socially isolated to comply with governmental recommendations. These results are consistent with findings in the international literature stating that people who are higher in neuroticism tend to avoid risks (Jonason and Sherman, 2020). Although our sample is composed of young adults, the ones who have higher levels of neuroticism appeared to be more prone to worry about the social consequences of their behavior. This suggests that psychological characteristics, like personality traits or individual perceptions of risk, correlate with resilience to stressful personal and collective events. It also means that such traits have a social impact, as they manifest in pro-social behaviors and actions that benefit the collectivity.

Our data also reveal that the group that declared that they socially isolate to comply with governmental recommendations had higher levels of conscientiousness, mostly worked in the public sector, and are older. Those who have a more conservative profile seems to be associated with engagement in social isolation “to do the right thing” or what is socially expected, without questioning it (Zajenkowski, et al., 2020). In terms of mental health, these people reported less psychological distress, anxiety, depression and stress, negative affect, and more positive affect. In a sociological perspective, these results are aligned with the article by (Matthewman and Huppatz, 2020), entitled “A sociology of Covid-19”. For the authors, the Covid-19 pandemic provides opportunities for ‘disaster capitalists’ to profit and it will enhance certain forms of surveillance, and it will impact some groups far more negatively than others.

Results from Phase 2 present a different scenario and warrant attention, and a preamble on the current political and social situation in Brazil. In the first month of the pandemic, the health minister communicated with the public and provided guidelines and procedures to face the Covid-19 pandemic in a daily national report. Official media reported daily the number of people affected by Covid-19, the number of deaths, and statistics on the spread of the virus. It was made clear what actions were expected of individuals to be done and what each Brazilian region was facing and should do. However, because of political factions and internecine tension in the political establishment, this flow of data and information was put to a halt. The health minister was replaced more than once, and in the third month of the pandemic there was no health minister to lead the management and response to the pandemic. This situation still persists despite evidence and research from multiple disciplines that highlights the importance of production and access to transparent information and of governmental leadership to face better adversity such as the current one.

So, in the first month of the pandemic in Brazil we conclude that people who reported that they engaged in social isolation to comply with governmental recommendations may have also had less psychological distress and negative health outcomes. In fact, they were told what to do; they had a clearly expected behaviors to follow. The stress they were facing could have been positive (stress mindset), and despite the stressful situation they were living, it was possible for them to still experience positive affect. This interpretation of the results aligns with the argument of (Lazarus and Folkman, 1984). According to their coping theory when individuals appraised the stress as a challenge, they are more prone to use problem focused coping strategies and got more positive health outcomes.

By the third month of the pandemic in Brazil the recommendations from the government became unclear and were highly inconsistent (Aquino et al., 2020; Moreira, 2020). We observed in our data an increase in psychological distress for this group (people who engaged in social isolation to comply with governmental recommendations). This can be partially explained by a phenomenon that, in the macrocontext, (Joia and Michelotto, 2020), explained as universalism x utilitarianism. The authors pointed out that the pandemic made explicit that the Brazilian population is divided into two contrasting philosophical approaches: the universalism—understanding life as an asset of infinite value and, therefore, more important than the country’s economic preservation—and the utilitarianism—where the focus is on the mitigation of the Covid 19 pandemic-enabled economic crisis. The main cause for these different sense-makings is associated with the lack of a monosemic definition for the Covid-19 pandemic construct. The authors add that trends emphasized by experts, such as a new-normal and the digital transformation of society, played a peripheral role in the social representation of the Covid 19 pandemic in Brazil. So, for the ones who were following the governmental recommendations, which have prioritize since the begging of the pandemic the economic aspect, the lack of recommendation plus the increase of economic problems caused by the pandemic increase the psychological distress.

This paper described some personality traits that fit the universalist profile, and others the utilitarian postulated by (Joia and Michelotto, 2020). In this way it can help to understand the macro category of Brazilian social reality nowadays and the way the brazilian Society are facing the Covid pandemic.

### Limitations and Further Research

We recognize the limitation of using the internet and social media or social networks for the recruitment of the participants and for the administration of the questionnaire. In particular, we note the drop in response rate from one phase to the next. In phase 2, the respondents were less than a third than phase 1. In these few months, people were overstudied, and constantly solicited to respond to a variety of internet surveys on all aspects of the Covid-19 pandemic. The proliferation of surveys and assessments unfortunately has not been accompanied by the application of rigor and scientific standards of quality. Recent studies remind us of the need to balance between the easy and quick access to data worldwide via internet surveys and the slower and rigorous process that prevents cumulation of misunderstanding and research malpractices (Dinis-Oliveira, 2020) or sample bias (Nikolov et al., 2015).

As mentioned before, Brazil is a big country with great social disparities what can be observed also in the access to the internet, to information, and to health services (Barros et al., 2000). These disparities produce substantial bias, especially in data collection mediated by the internet. This is obvious in our study; compliance with social isolation, whatever the motive, was reported by about 95% of the respondents. In both phases, the average age was 34, and the sample was composed mostly of single women and professionals, undergraduate and graduate level of education. Also, the authors of this manuscript are university professors who utilized their social networks to circulate the links to social media and for engaging respondents in the survey (Nikolov et al., 2015). However, although all these observations must be taken into account they highlighted once more the necessity of accessing psychosocial aspects to understand better social behaviors especially in complex societies like Brazil.

## Conclusion

This study shows that in phase 1, people who practiced social isolation to comply with governmental recommendations reported less psychological distress, anxiety, depression and stress, negative affect and more positive affect than people with other motives. But that result was not reproduced in phase 2 three months after the pandemic began in Brazil. The data point to the importance of considering the psychological characteristics and their influence on the social behavior of individuals and on supportive (pro-social) behavior, even in a period of significant stress and high social risk.

In terms of personality traits, the group which socially isolated to avoid infecting others was also the one which has higher neuroticism levels and less conscientiousness levels compared to those who practiced social isolation to comply with governmental recommendations. This finding is consistent with research on people who are higher in neuroticism and who also tend to avoid risks (Jonason and Sherman, 2020). Our results remind of the importance of considering psychological traits and their influence on individuals’ social and pro-social behavior, even in a period of significant stress and high social risk.

According to Singu et al. (2020), studying the social determinants of health, and how they impact populations during times of crisis, will help governments to manage better health emergencies. This seems to be particularly important in the case of Brazil, a country of continental dimensions and with great economic inequality. We argue that government actions based on empirical evidence are particularly important.

Our results can support the development of public policies. However, it is important to highlight that Brazil is a new nation, in all senses, including the maturity of its public policies as actions and services that serve the citizen as a subject of rights. In Brazil, thinking about coping with this social, political, economic and culturally constituted reality is to conceive a set of structuring public policies, capable of acting intersectorally and jointly with the purpose of guaranteeing citizens access to all legally constituted rights. Social policies should not be operationalized without considering their social context, their intervention reality, and in the case of Brazil, their context of poverty and social inequality (Godinho, 2011).

## Data Availability

The raw data supporting the conclusions of this article will be made available by the authors, without undue reservation.
